# Association of ABO and Rh blood groups with clinicopathological features and lymph node involvement in surgically treated thyroid malignancies: a retrospective single-center study

**DOI:** 10.1186/s12902-026-02433-5

**Published:** 2026-07-22

**Authors:** Ahmet Zahit Kaan, Murat Acar, Harun Çok, Serhan Yılmaz, Erkan Somuncu

**Affiliations:** 1 Department of General Surgery, Hakkari Derecik State Hospital, Hakkari, Türkiye; 2 Department of General Surgery, Muş Bulanık State Hospital, Muş, Türkiye; 3 Department of General Surgery, Istanbul Buyukcekmece Mimar Sinan State Hospital, Istanbul, Türkiye; 4 Department of General Surgery, Ankara Bilkent City Hospital, Ministry of Health, Ankara, Türkiye; 5 Department of General Surgery, Istanbul Kanuni Sultan Süleyman Training and Research Hospital, Istanbul, Türkiye

**Keywords:** ABO blood-group system, Rh-Hr blood-group system, Thyroid neoplasms, Lymph node metastasis, Clinicopathological features, Prognostic factors, Thyroidectomy

## Abstract

This study investigated the association between ABO and Rh blood group antigens and the clinicopathological features of 418 patients who underwent thyroidectomy for primary thyroid malignancy between December 2016 and June 2023. Since continuous variables did not follow a normal distribution (*p* < 0.05), data were presented as median and interquartile range (IQR). The distribution of ABO blood groups was A (44.5%), O (31.8%), B (16.0%), and AB (7.7%), with 90.9% of patients being Rh-positive. The median age of the cohort was 48 years (IQR: 19). While ABO groups showed no significant difference in age, Rh-negative patients were significantly older than Rh-positive patients (52.5 vs. 48.0 years; p = 0.027). No direct association was identified between ABO groups and the overall clinicopathological features such as tumor size or multifocality (p > 0.05). However, significant differences were observed regarding pathologically confirmed lymph node (LN) involvement. Pathologically confirmed LN involvement was significantly more prevalent in the Rh-negative group compared to the Rh-positive group (15.8% vs. 5.5%; p = 0.027). Most notably, a subgroup analysis revealed that patients with the AB blood group had a significantly higher rate of pathologically confirmed LN involvement compared to non-AB groups (15.6% vs. 5.7%; p = 0.028). To confirm the independence of this association, a multivariable logistic regression analysis was performed. After adjusting for potential confounders including age and tumor size, the AB blood group was independently associated with pathologically confirmed LN metastasis in an exploratory multivariable model (OR: 3.114, 95% CI: 1.085–8.938, p = 0.035). These findings suggest that while ABO and Rh blood groups are not significantly associated with most clinicopathological variables in this cohort, they demonstrate specific correlations with nodal disease. These findings may represent exploratory signals that warrant further validation, rather than clinically applicable biomarkers at this stage. Further multicenter prospective studies are warranted to validate these associations across all histological subtypes.

## Trial registration

Not applicable.

## Introduction

Thyroid cancer is the most common endocrine malignancy, and its incidence has been steadily increasing worldwide. According to the World Health Organization (WHO) 2022 data, it ranks as the 5th most common malignancy in Turkey, with an incidence of 18 per 100,000 [1, 2]. Although most thyroid nodules are asymptomatic, approximately 4% to 6.5% of clinical nodules are malignant, with higher risks observed in children, adults under 30 or over 60 years of age, and individuals with a family history of the disease [3, 4] Among these malignancies, Papillary Thyroid Carcinoma (PTC) is the most frequent histological subtype. While the prognosis is generally favorable, advanced age (> 55) and regional or distant metastasis significantly influence clinical outcomes [5]. Rare but aggressive types, such as Anaplastic Thyroid Cancer (ATC), carry a poor prognosis, with most cases presenting at an advanced stage [6]. Additionally, Medullary Thyroid Cancer (MTC) is characterized by early lymph node metastasis and requires distinct surgical and follow-up protocols [7].

The ABO and Rhesus (Rh) systems are the primary antigenic markers on the erythrocyte surface. Beyond transfusion medicine, specific blood groups have been associated with various malignancies, such as the link between group A and gastric cancer or non-O groups and pancreatic cancer [8]. However, studies investigating the association between ABO/Rh antigens and the clinicopathological behavior of thyroid cancer remain limited in the literature. The aim of the present study was to evaluate the association between ABO and Rh blood groups and clinicopathological features, with a specific focus on lymph node involvement, in a cohort of patients undergoing surgery for thyroid malignancy. By identifying potential biological markers, we aim to contribute to the understanding of regional metastasis risk and identify patient subgroups that may necessitate closer clinical follow-up.

## Materials and methods

This retrospective cohort study was conducted at the General Surgery Clinic of Istanbul Kanuni Sultan Süleyman Training and Research Hospital, following approval from the local Ethics Committee (Approval No: 2023.09.115). The study was performed in accordance with the ethical standards of the 1964 Declaration of Helsinki and its subsequent amendments. Initially, 1188 patients who underwent thyroidectomy between December 2016 and June 2023 were screened for eligibility. The inclusion criteria were defined as: (i) aged 18 years or older, (ii) a final histopathological diagnosis of primary thyroid malignancy, (iii) documented ABO and Rh blood group data, and (iv) no prior history of neck surgery. To ensure a scientifically robust analysis and minimize selection bias, a rigorous exclusion process was implemented; 769 patients with benign pathologies (e.g., multinodular goiter, thyroiditis) and one patient with non-thyroid primary malignancy were excluded. Consequently, a final cohort of 418 patients who met all criteria was included in the final analysis.

Data retrieved from medical records included demographics (age, gender, BMI), ABO/Rh blood groups, and preoperative laboratory parameters (TSH, fT4, fT3, Tg). Ultrasonography (USG) findings, such as nodule size, number, calcification, and Bethesda classification, were systematically recorded. Detailed histopathological reports were analyzed for tumor size, histological type, multifocality, capsule invasion, extrathyroidal extension, and lymph node involvement. Lymph node (LN) involvement was strictly defined as pathologically confirmed nodal metastasis in patients who underwent lymph node dissection (N1 status). To maintain statistical power, only parameters consistently available for all 418 patients were included in the final models.

Statistical analyses were performed using SPSS version 22.0 (SPSS Inc., Chicago, IL, USA). Categorical variables are presented as frequencies and percentages. The normality of continuous variables, such as age and tumor size, was assessed using the Shapiro-Wilk test. As these variables did not follow a normal distribution, they are presented as median and interquartile range (IQR). For comparisons between independent groups, the Kruskal-Wallis test was used for ABO blood groups, and the Mann-Whitney U test was used for Rh antigens. Categorical data were compared using the Chi-Square test, or Fisher’s Exact test for cross-tabulations with small expected cell counts (< 5). Patients who did not undergo formal lymph node dissection were classified as having no pathologically confirmed nodal metastasis recorded for the purpose of the statistical models. Furthermore, to control for potential confounding factors and evaluate independent associations with pathologically confirmed lymph node involvement, an exploratory multivariable logistic regression analysis was performed. The multivariable logistic regression model incorporated the AB blood group, age, and tumor size. Age and tumor size were selected as covariates because they are well-established, independent prognostic factors for thyroid cancer aggressiveness, allowing us to assess if blood group status remains independently associated with nodal disease. Results are reported as odds ratios (OR) with 95% confidence intervals (CI), and a p-value of < 0.05 was considered statistically significant.

## Results

A total of 418 patients were included in the final analysis, consisting of 348 (83.3%) females and 70 (16.7%) males. The distribution of ABO blood groups was as follows: group A (44.5%), group O (31.8%), group B (16.0%), and group AB (7.7%). Regarding the Rhesus system, 380 (90.9%) patients were Rh-positive and 38 (9.1%) were Rh-negative. Since continuous variables did not follow a normal distribution (*p* < 0.05), they are presented as median and interquartile range (IQR). The median age of the cohort was 48 years (IQR: 19; range: 18–81). Preoperative median TSH and fT4 levels were recorded as 1.52 mIU/L (IQR: 1.56) and 1.21 ng/dL (IQR: 0.27), respectively. Detailed clinical and demographic characteristics are summarized in Table 1.

**Table 1:** Distribution of clinicopathological data of the patients

		Number	%
Gender	Female	348	83,3
	Male	70	16,7
ABO Blood Group	A	186	44,5
	B	67	16
	0	133	31,8
	AB	32	7,7
Rh Blood Group	Positive	380	90,9
	Negative	38	9,1
Nodule Diameter (on USG)	< 4 cm	356	85,2
	> 4 cm	62	14,8
Nodule Number (on USG)	Solitary	232	55,5
	Multiple	186	43,5
Presence of Calcification (on USG)	Present	236	56,5
	Absent	182	43,5
Bethesda Classification	Non-diagnostic	33	7,9
	Benign	63	15,1
	Atypia of Undetermined Significance	95	22,7
	Follicular Neoplasm	4	1
	Suspicious for Malignancy	91	21,8
	Malignant	132	31,6
Surgical Procedure	Total Thyroidectomy	373	89,2
	Lobectomy	45	10,8
Pathology Result	Papillary (Carcinoma)	393	94
	Follicular (Carcinoma)	11	2,6
	Medullary (Carcinoma)	14	3,3
Multifocality	Absent	251	60
	Present	167	40
Extrathyroidal Extension	Absent	367	87,7
	Present	51	12,2
Capsule Invasion	Absent	368	88
	Present	50	12
Vascular Invasion	Absent	350	83,7
	Present	68	16,3
Lymphatic Invasion	Absent	342	81,8
	Present	76	18,2
Lymph Node Involvement	Absent	391	93,5
	Present	27	6,5
Tumor Diameter	< 1 cm	179	42,8
	1–2 cm	136	32,5
	2–4 cm	72	17,2
	> 4 cm	31	7,4
Lymph Node Dissection	Absent	353	84,4
	Central only	43	10,3
	Central and Lateral	22	5,3

Ultrasonographic (USG) evaluation revealed a median nodule size of 18.5 mm (IQR: 24). Solitary nodules were present in 232 (55.5%) patients, and calcification was observed in 236 (56.5%). According to the Bethesda classification of FNAB results, 132 (31.6%) cases were malignant and 91 (21.8%) were suspicious for malignancy. Total thyroidectomy was performed in 373 (89.2%) patients, while 45 (10.8%) underwent lobectomy. Postoperative pathological examination showed a median tumor size of 12 mm (IQR: 13). Among patients who underwent lymph node dissection, a median of 12 (IQR: 25) lymph nodes were excised. Postoperative median TSH and fT4 levels were 3.52 mIU/L (IQR: 22.81) and 1.14 ng/dL (IQR: 0.72), respectively.

No statistically significant differences were observed between ABO blood groups regarding age (*p* = 0.349), preoperative/postoperative thyroid functions, nodule size (*p* = 0.097), or tumor size (*p* = 0.932). Furthermore, clinicopathological features—including gender, USG calcification, Bethesda scores, multifocality, capsule invasion, extrathyroidal extension, and vascular/lymphatic invasion—did not differ significantly across ABO blood groups (*p* > 0.05). Comparisons of group O vs. non-O, A vs. non-A, and B vs. non-B also yielded no significant associations across the analyzed parameters.

Significant associations were identified regarding the Rhesus (Rh) system. The median age was significantly higher in Rh-negative patients compared to the Rh-positive group (52.5 vs. 48.0 years, respectively; *p* = 0.027). Furthermore, pathologically confirmed lymph node involvement was significantly more frequent in the Rh-negative group compared to the Rh-positive group (15.8% vs. 5.5%; *p* = 0.027), as summarized in Table 2. Notably, a subgroup analysis comparing the AB blood group with non-AB groups revealed that pathologically confirmed lymph node involvement was significantly higher in patients with the AB blood group (15.6% vs. 5.7%; *p* = 0.028; Table 3). To confirm the independence of this association, a multivariable logistic regression analysis was performed. After adjusting for potential confounders, including age and tumor size, the AB blood group was independently associated with pathologically confirmed lymph node metastasis in this exploratory multivariable model (OR: 3.114, 95% CI: 1.085–8.938, *p* = 0.035), as detailed in Table 4.

**Table 2:** Comparison of patients’ clinicopathological features with Rh blood groups

		Blood Groups				*p*
		Rh +		Rh -		
		Number	%	Number	%	
Gender	Female	317	83,4	31	81,6	0,772
	Male	63	16,6	7	18,4	
Nodule Diameter (on USG)	<4 cm	320	84,2	36	94,7	0,095
	>4 cm	60	15,8	2	5,3	
Nodule Number (on USG)	Solitary	210	55,3	22	57,9	0,864
	Multiple	170	44,7	16	42,1	
Presence of Calcification (on USG)	Absent	165	43,4	17	44,7	0,866
	Present	215	56,6	21	55,3	
Bethesda Classification	Non-diagnostic	32	8,4	1	2,6	0,529
	Benign	56	14,7	7	18,4	
	Atypia of Undetermined Significance	89	23,4	6	15,8	
	Follicular Neoplasm	4	1,1	0	0	
	Suspicious for Malignancy	80	21,1	11	28,9	
	Malignant	119	31,3	13	34,2	
Surgical Procedure	Total Thyroidectomy	339	89,2	34	89,5	0,662
	Lobectomy	41	10,8	4	10,5	
Pathology Result	Papillary (Carcinoma)	356	93,7	37	97,4	0,545
	Follicular (Carcinoma)	11	2,9	0	0	
	Medullary (Carcinoma)	13	3,4	1	2,6	
Multifocality	Absent	226	59,5	25	65,8	0,491
	Present	154	40,5	13	34,2	
Capsule Invasion	Absent	334	87,9	34	89,5	1,0
	Present	46	12,1	4	10,5	
Extrathyroidal Extension	Absent	335	88,2	32	84,2	0,441
	Present	45	11,8	6	15,8	
Vascular Invasion	Absent	332	84,7	28	73,7	0,103
	Present	58	15,3	10	26,3	
Lymphatic Invasion	Absent	315	82,9	27	71,1	0,079
	Present	65	17,1	11	28,9	
Lymph Node Involvement	Absent	359	94,5	32	84,2	**0,027**
	Present	21	5,5	6	15,8	
Tumor Diameter	<1 cm	162	42,6	17	44,7	0,944
	1-2 cm	123	32,4	13	34,2	
	2-4 cm	66	17,4	6	15,8	
	>4 cm	29	7,6	2	5,3	
Lymph Node Dissection	Absent	322	84,7	31	81,6	0,290
	Central only	40	10,5	3	7,9	
	Central and Lateral	18	4,7	4	10,5	

Data analyzed using Chi-square test or Fisher’s exact test where appropriate

**Table 3:** Comparison of patients’ clinicopathological features across AB and non-AB blood groups

		AB		Non-AB		p	
		Number	%	Number %			
Gender	Female	28	83,3	320	82,9		0,503
	Male	4	16,7	66	17,1		
Nodule Diameter (on USG)	< 4 cm	28	87,5	328	85,0		0,699
	> 4 cm	4	12,5	58	15,0		
Nodule Number (on USG)	Solitary	18	56,3	214	55,4		0,929
	Multiple	14	43,8	172	44,6		
Presence of Calcification (on USG)	Absent	18	56,3	164	42,5		0,131
	Present	14	43,8	222	57,5		
Bethesda Classification	Non-diagnostic	2	6,3	31	8,0		0,528
	Benign	7	21,9	56	14,5		
	Atypia of Undetermined Significance	4	12,5	91	23,6		
	Follicular Neoplasm	0	0	4	1,0		
	Suspicious for Malignancy	6	18,8	85	22,0		
	Malignant	13	40,6	119	30,8		
Surgical Procedure	Total Thyroidectomy	30	93,7	343	88,9		0,617
	Lobectomy	2	6,3	43	11,1		
Pathology Result	Papillary (Carcinoma)	29	90,6	364	94,3		0,623
	Follicular (Carcinoma)	1	3,1	10	2,6		
	Medullary (Carcinoma)	2	6,3	12	3,2		
Multifocality	Absent	17	53,1	234	60,6		0,405
	Present	15	46,9	152	39,4		
Capsule Invasion	Absent	27	84,4	341	88,3		0,506
	Present	5	15,6	45	11,7		
Extrathyroidal Extension	Absent	25	78,1	342	81,8		0,082
	Present	7	21,9	44	11,4		
Vascular Invasion	Absent	24	75,0	326	84,5		0,164
	Present	8	25,0	60	15,5		
Lymphatic Invasion	Absent	26	81,3	316	81,9		0,931
	Present	6	18,8	70	18,1		
Lymph Node Involvement	Absent	27	84,4	364	94,3		**0**,**028**
	Present	5	15,6	22	5,7		
Tumor Diameter	< 1 cm	13	40,6	166	43,0		0,937
	1–2 cm	12	37,5	124	32,1		
	2–4 cm	5	15,6	67	17,4		
	> 4 cm	2	6,3	29	7,5		
Lymph Node Dissection	Absent	24	75,0	329	85,2		0,241
	Central only	6	18,8	37	9,6		
	Central and Lateral	2	6,3	20	5,2		

Data analyzed using Chi-square test or Fisher’s exact test where appropriate

**Table 4:** Multivariable logistic regression analysis of predictors for lymph node metastasis

	B	S.E.	Wald	df	Sig.	OR	95% CI	
							Lower	Upper
AB Blood Group	1,136	0,538	4,459	1	**0**,**035**	3,114	1,085	8,938
Age	-0,023	0,017	1,89	1	0,169	0,977	0,946	1,01
Tumor Size	0,013	0,012	1,209	1	0,272	1,013	0,99	1,038
Constant	-1,961	0,81	5,861	1	0,015	0,141		

B: Coefficient; S.E.: Standard Error; Sig.: Statistical significance; OR: Odds Ratio; CI: Confidence Interval. *p* < 0.05

## Discussion

The relationship between blood group antigens and cancer has been a subject of long-standing interest, with numerous studies investigating potential associations across various malignancies. While blood group A has been linked to higher incidences of gastric and pancreatic cancers, blood group O is often associated with a reduced risk [9–11]. Similarly, a study by Ürün et al. reported an increased risk of lung cancer in patients with non-O blood groups and Rh-negative status [12]. Many studies similar to these can be found in the literature [13–16]. Meta-analyses have further suggested that blood group A may increase the risk of breast and ovarian cancers, whereas blood group O appears protective across multiple organ systems [17]. Although the exact biological mechanisms remain to be fully elucidated, hypotheses suggest that ABO glycosyltransferase activity may influence tumor-promoting molecules or that genetic variations at the ABO locus may alter plasma concentrations of adhesion molecules like E-selectin and ICAM-1, which play pivotal roles in inflammation and immune cell adhesion [18].

Despite the rising global incidence of thyroid cancer, literature regarding its association with ABO and Rh blood groups remains limited and contradictory. The distribution of ABO and Rh antigens in our cohort (A: 44.5%, O: 31.8%, B: 16%, AB: 7.7%; Rh-positive: 90.9%) was largely consistent with established epidemiological data for the Turkish population [19]. While some studies, such as those by Doğan et al., found no correlation between blood groups and clinicopathological variables in thyroid cancer, our study identified a significant association between Rh status and age [20]. Specifically, Rh-negative patients in our cohort had a significantly higher median age (52.5 vs. 48.0 years; *p* = 0.027).

Our findings regarding the lack of a significant association between ABO blood groups and most clinicopathological variables in this cohort align with the results of Tam et al. [21]. However, the most striking finding of our study was the relationship between specific blood groups and lymph node involvement. While some literature, such as the work of Oroei et al., focuses on incidence rates, our data points toward the prognostic value of blood antigens [22]. We initially observed that pathologically confirmed lymph node involvement was significantly higher in patients with the AB blood group compared to non-AB groups (15.6% vs. 5.7%; *p* = 0.028). To rigorously validate this observation, we performed a multivariable logistic regression analysis adjusting for potential confounders such as age and tumor size. Crucially, the AB blood group was independently associated with pathologically confirmed lymph node metastasis in this exploratory multivariable model, carrying a 3.1-fold increased risk (OR: 3.114, 95% CI: 1.085–8.938, *p* = 0.035). This indicates that the association is not merely a reflection of age or tumor-related differences but is independently linked to the AB blood group status, suggesting a potentially more aggressive regional spread in these patients. While recent studies, such as those by Dogan [20] and Ghunaim [23], have evaluated ABO blood group distributions in thyroid cancer patients, our study differentiates itself by specifically focusing on lymph node metastasis as a primary prognostic outcome within a surgically treated cohort.

Furthermore, we identified a significant relationship between Rhesus status and nodal metastasis. Pathologically confirmed lymph node involvement was significantly more prevalent in the Rh-negative group compared to the Rh-positive group (15.8% vs. 5.5%; *p* = 0.027). This aligns with findings by Kamacı et al., who also reported higher rates of lymph node involvement in Rh-negative patients [24]. Although other studies have suggested links between blood group B and extrathyroidal invasion or group AB and vascular invasion, we did not find statistically significant associations with these specific features [21, 23].

Although these findings suggest an association between specific blood groups and pathologically confirmed nodal involvement, they should be interpreted as exploratory and hypothesis-generating rather than a direct clinical indication to alter the surgical threshold for lymph node dissection. While these associations are statistically significant in our multivariable model, they are not yet sufficient to mandate changes in clinical guidelines or direct surgical decision-making without further robust, prospective evidence.

However, this study has several limitations that must be addressed. First, its retrospective and single-center nature may limit the generalizability of the findings. Furthermore, while our study included various primary thyroid malignancies, the vast majority of the cohort consisted of papillary thyroid carcinoma (PTC); therefore, the results should be interpreted with caution regarding other histological subtypes. Second, the sample sizes for the AB blood group (*n* = 32) and Rh-negative patients (*n* = 38) were relatively small; therefore, these findings should be interpreted as strong exploratory signals that warrant further validation. Furthermore, since the multivariable logistic regression model incorporated a relatively small number of pathologically confirmed nodal metastasis events (*n* = 27), the risk of model overfitting remains an important limitation. This limited event-per-variable ratio may directly affect the precision and stability of the reported odds ratio for the AB blood group (OR: 3.114), necessitating a cautious interpretation. Third, as 84.4% of the cohort did not undergo formal lymph node dissection, a significant detection bias exists. This means our primary outcome measure reflects pathologically confirmed disease rather than the true biological prevalence of metastasis in the entire cohort. Additionally, the risk of Type I error (false-positive results) remains, as multiple subgroup comparisons were performed without formal correction methods like Bonferroni or FDR. Finally, due to the excellent prognosis of the cohort and the lack of long-term follow-up, survival analyses were not performed. Further prospective, multicenter studies with larger cohorts and detailed genetic analyses are required to clarify the underlying biological mechanisms.

## Conclusion

In conclusion, our study indicates that while ABO and Rh blood group systems are not significantly associated with most clinicopathological variables in this cohort, they demonstrate specific correlations. Our findings highlight that Rh-negative status is associated with an older age at diagnosis and a higher prevalence of pathologically confirmed lymph node metastasis. Most importantly, our multivariable analysis revealed that the AB blood group is independently associated with increased pathologically confirmed regional nodal involvement in this exploratory multivariable model (OR: 3.114, *p* = 0.035), even after adjusting for potential confounders such as age and tumor size. Since our cohort primarily consisted of papillary thyroid carcinoma (PTC), these findings may represent exploratory signals that warrant further validation, rather than clinically applicable biomarkers at this stage. However, given the exploratory nature of these findings and the retrospective design of our study, these results should be considered strictly hypothesis-generating and are not yet sufficient to mandate changes in clinical surgical protocols. Further large-scale, multicenter prospective studies are necessary to validate these associations and elucidate the underlying biological mechanisms.

## Data Availability

The datasets generated during and/or analyzed during the current study are available from the corresponding author on reasonable request.
